# Structure and Emerging Functions of LRCH Proteins in Leukocyte Biology

**DOI:** 10.3389/fcell.2020.584134

**Published:** 2020-09-22

**Authors:** Thibaud Rivière, Almke Bader, Kristin Pogoda, Barbara Walzog, Daniela Maier-Begandt

**Affiliations:** ^1^Institute of Cardiovascular Physiology and Pathophysiology, Biomedical Center, Ludwig-Maximilians-Universität München, Munich, Germany; ^2^Walter Brendel Center of Experimental Medicine, University Hospital, Ludwig-Maximilians-Universität München, Munich, Germany; ^3^Department of Physiology, Medical Faculty, Augsburg University, Augsburg, Germany

**Keywords:** immunity, leukocyte, trafficking, migration, LRCH, actin

## Abstract

Actin-dependent leukocyte trafficking and activation are critical for immune surveillance under steady state conditions and during disease states. Proper immune surveillance is of utmost importance in mammalian homeostasis and it ensures the defense against pathogen intruders, but it also guarantees tissue integrity through the continuous removal of dying cells or the elimination of tumor cells. On the cellular level, these processes depend on the precise reorganization of the actin cytoskeleton orchestrating, e.g., cell polarization, migration, and vesicular dynamics in leukocytes. The fine-tuning of the actin cytoskeleton is achieved by a multiplicity of actin-binding proteins inducing, e.g., the organization of the actin cytoskeleton or linking the cytoskeleton to membranes and their receptors. More than a decade ago, the family of leucine-rich repeat (LRR) and calponin homology (CH) domain-containing (LRCH) proteins has been identified as cytoskeletal regulators. The LRR domains are important for protein-protein interactions and the CH domains mediate actin binding. LRR and CH domains are frequently found in many proteins, but strikingly the simultaneous expression of both domains in one protein only occurs in the LRCH protein family. To date, one LRCH protein has been described in drosophila and four LRCH proteins have been identified in the murine and the human system. The function of LRCH proteins is still under investigation. Recently, LRCH proteins have emerged as novel players in leukocyte function. In this review, we summarize our current understanding of LRCH proteins with a special emphasis on their function in leukocyte biology.

## Introduction

Leukocytes constantly patrol within the circulation and tissue to ensure the defense against pathogen intruders and to guarantee tissue integrity through the continuous removal of dying cells and the elimination of tumor cells (Ostrand-Rosenberg, 2008; Nourshargh and Alon, 2014). Proper immune cell function fundamentally depends on the highly dynamic regulation of the actin cytoskeleton (Moulding et al., 2013; Janssen and Geha, 2019). On the cellular level, this regulation involves actin-binding proteins as well as their effector proteins that respond to signals transduced by e.g., immunoreceptors, chemokine receptors and integrins (Mocsai et al., 2015; Begandt et al., 2017; Zehrer et al., 2018). Hence, fine-tuning of actin polymerization, branching and tethering to membranes and receptors enables, e.g., leukocyte polarization and migration and mediates vesicular dynamics important for cytokine release or phagocytosis. Within the plethora of actin-binding and regulatory proteins, a few have already been described to tremendously affect leukocyte function, such as Wiskott-Aldrich syndrome protein (WASp), mammalian actin-binding protein 1, coronin 1A (Coro1A), filamin A (FLNa), and dedicator of cytokinesis 8 (DOCK8) (Snapper et al., 2005; Schymeinsky et al., 2009; Harada et al., 2012; Pick et al., 2017; Roth et al., 2017). Thereby, the protein binding to actin is facilitated by many different domains with one of the most common being the calponin homology (CH) domain as in FLNa (Gimona et al., 2002; Yin et al., 2020). Another family is represented by the leucine-rich repeat (LRR) and CH domain-containing (LRCH) proteins which are specifically interesting for leukocyte biology as many proteins important for innate immunity contain LRR domains (Alder et al., 2005; Istomin and Godzik, 2009; Ng et al., 2011). The occurrence of both domains in the same protein is only known for the LRCH protein family (Foussard et al., 2010). To date, one LRCH protein has been described in *Drosophila melanogaster* and four LRCH proteins have been identified in mice, rats and humans. The function of LRCH proteins is still enigmatic but they have been suggested to act as cytoskeletal regulators (Foussard et al., 2010). In this review, we provide a comprehensive overview on the current understanding of the structure and function of LRCH proteins with a special emphasis on leukocyte biology.

## Structure of LRCH Proteins

**FIGURE 1:**
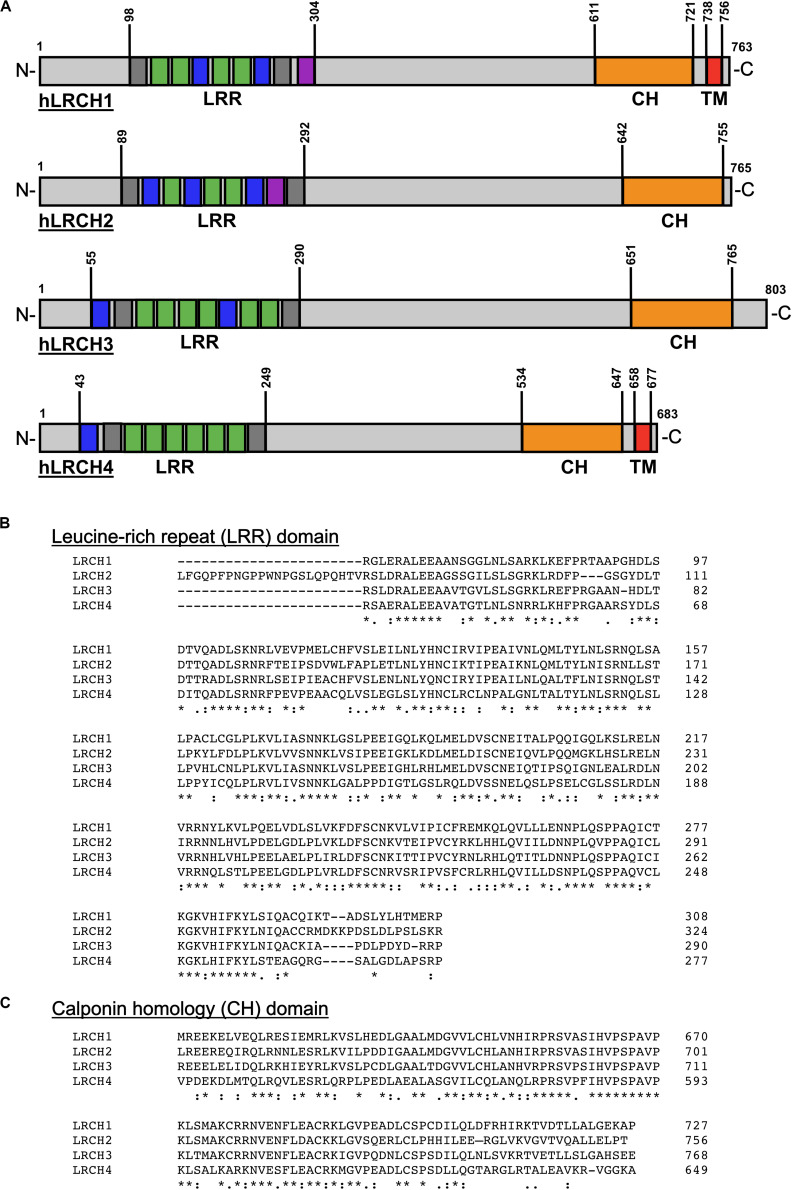
Structure and sequence of the human LRCH family proteins. **(A)** Leucine-rich repeat (LRR), calponin homology (CH) and transmembrane (TM) domains in human (h)LRCH proteins1–4. LRR classes are color-coded (plant-specific: green, irregular: gray, bacterial: blue, SDS22: purple). Numbers label amino acid residues. **(B,C)** Sequence alignment of human LRR **(B)** and CH **(C)** domains using ClustalOmega (Madeira et al., 2019). Identical (*), strongly similar (:) and weakly similar (.) amino acid residues are indicated below the alignment.

The structure of the four members of the human (h)LRCH proteins characterized by an N-terminal LRR domain and a C-terminal CH domain is shown in Figure 1A (Foussard et al., 2010). In addition to the unique combination of LRR and CH domains, a putative transmembrane (TM) domain within the C-terminus has been described for LRCH1 and LRCH4 (Ng et al., 2011; Aloor et al., 2019). Sequence alignments of all four human LRCH proteins revealed modest sequence identity ranging between 33.1 and 39.4%. When comparing specifically the LRR and CH domains, the sequence identity increases up to 70.0% and the sequence similarity up to 85.0%, implying that the LRCH proteins may show some functional redundancy (Figures 1B,C).

### LRR Domain

LRR domains consist of a chain of two to 42 LRRs with a single LRR commonly being 20–30 amino acids long (Enkhbayar et al., 2004). In general, the LRR domain of LRCH proteins in drosophila, mice, rats and humans consists of nine LRRs. Only the LRR domains of LRCH1 in rats and LRCH3 in humans, mice and rats contain 10 LRRs (Figures 1A,B). Based on their sequence, the LRRs are divided into eight classes: bacterial (S), ribonuclease inhibitor-like (RI), cysteine-containing (CC), SDS22, plant-specific (PS), typical (T), *Treponema pallidum* (Tp), and irregular. In humans, all LRCH proteins contain LRRs of the S, PS, and irregular class. Further, hLRCH1, hLRCH2, and hLRCH3 contain SDS22 class LRRs (Kajava et al., 1995; Kajava, 1998; Ng et al., 2011). The types of LRR in drosophila, murine, and rat LRCH proteins have not been classified yet. The LRR domain forms a horseshoe-shaped, superhelical arrangement with the LRRs as repeating structural units that enables the domain to participate in a large array of protein-protein interactions, including dimerization of LRR-containing proteins (Liu et al., 2008, 2020; Wang et al., 2010; Afzal et al., 2013). The exact function of most LRR-containing proteins, including LRCH proteins is still poorly understood. Interestingly, a remarkable number of proteins containing LRRs of the S or T class have important functions related to immunity and receptor-mediated signaling, such as Toll-like receptors (TLRs), suggesting an important role of LRCH proteins in immunity (Pålsson-McDermott and O’Neill, 2007; Ng et al., 2011).

### CH Domain

CH domains are approximately 100 amino acids long and are homologous to the N-terminal region of the cytoskeletal regulator calponin (Castresana and Saraste, 1995). CH domains of different proteins vary in their sequence but share a number of highly conserved core residues, resulting in a structural conservation of their globular shape (Ishida et al., 2008). Based on their sequence, CH domains are divided into several classes with the main classes being type 1–3 (Gimona et al., 2002; Yin et al., 2020). Generally, CH domains are able to interact with F-actin, although their actin-binding properties depend on their class affiliation. Type 1 and type 2 CH domains are typically organized in tandem, known to bind F-actin and thus found in cytoskeletal binding proteins, such as β-spectrin, plectin, or dystrophin (Gimona et al., 2002). Type 3 CH domains appear as single CH domain and are found in proteins with regulatory functions, such as calponin, Vav, and IQGAP1 (Gimona et al., 2002; Korenbaum and Rivero, 2002). Here, the CH domain of IQGAP1 binds to F-actin, whereas the CH domains of calponin and Vav are not involved in actin binding, but instead bind extracellular regulated kinase (ERK) or lead to dimerization, respectively (Gimona and Mital, 1998; Leinweber et al., 1999; Mateer et al., 2004). LRCH proteins contain a single type 3 CH domain located at the C-terminus (Figures 1A,C) but functional studies on the specific involvement of the CH domain in actin binding are still missing to date.

### Transmembrane Domain

LRCH proteins exist in different isoforms with two of three hLRCH1 and the longest isoforms of hLRCH4 and murine (m)LRCH4 presenting a putative TM domain at their C-terminus (Ng et al., 2011; Aloor et al., 2019). A search using the Phobius software for prediction of transmembrane topology^1^ further revealed the existence of a putative TM domain in the longest isoform of hLRCH3 and rat LRCH4, but experimental evidence for this observation is missing (Käll et al., 2007). So far only one study by Aloor et al. (2019) has specifically investigated mLRCH4 isoforms with and without TM domains and identified a critical role only for the TM domain-containing mLRCH4 for LPS binding and LPS-induced signal transduction. Thus, the presence of a TM domain may induce a functional change of LRCH proteins from signaling or scaffold proteins to proteins that sense external stimuli as the presence of a TM domain results in the extracellular localization of the LRR and CH domains.

## Genetic Analyses and Expression of LRCH Proteins

### Genomic Analyses

Genomic analyses of single nucleotide polymorphisms (SNPs) point toward a link between SNPs in LRCH proteins and different diseases. SNPs in *LRCH1* have been associated with a higher risk to develop osteoarthritis, although not all studies could confirm these findings (Spector et al., 2006; Snelling et al., 2007; Jiang et al., 2008; Panoutsopoulou et al., 2017). SNPs in *LRCH1* are also allocated to an increased risk of delayed encephalopathy after acute carbon monoxide poisoning (Gu et al., 2019). Pigs with SNPs in *LRCH3* are more susceptible to infections with *Escherichia coli* (*E. coli*) and cows with SNPs in *LRCH3* have a higher susceptibility to *Mycobacterium avium* ssp. *paratuberculosis* infection (Jacobsen et al., 2010; McGovern et al., 2019). Furthermore, gene amplifications of *LRCH2* and *LRCH3* are associated with tumorigenesis of low grade gliomas and melanomas, respectively (Liu et al., 2019; Williams et al., 2020).

### Transcriptional Analyses in Human Diseases

Strikingly, *LRCH* mRNA levels are regulated under inflammatory conditions. In the colonic mucosa of ulcerative colitis patients, *LRCH1* expression is markedly decreased and disease severity more pronounced with lower levels of *LRCH1* expression (Wang et al., 2020). In colorectal cancer, a potential complication of ulcerative colitis, expression of *LRCH3* is increased compared to colorectal tissues of healthy individuals and expression of *LRCH4* is increased in colorectal cancer patient samples with more advanced stages of cancer (Piepoli et al., 2012; Huo et al., 2017). In patients with acute myeloid leukemia, divided into low and high *LRCH4* expression groups, higher *LRCH4* expression is linked to decreased mortality (Sha et al., 2020). A significantly decreased *LRCH2* expression is found in breast cancer patients that developed brain metastasis compared to patients without brain metastasis or patients with primary brain tumors (Schulten et al., 2017).

### Transcriptional Analyses in Immune Responses

In leukocytes, *LRCH* expression is specifically regulated under inflammatory conditions. Monocyte-derived dendritic cells (DCs) stimulated with lipopolysaccharide (LPS) induce a negative feedback loop of cytokine production via microRNA-155 that affects *LRCH3* and *LRCH4* expression levels, which is in line with findings in HEK293 cells showing increased *LRCH3* levels upon stimulation with Epstein-Barr virus latent membrane protein (LMP1) (Ceppi et al., 2009; Gewurz et al., 2011). In human platelets exposed to *E. coli* K12 and RAW 264.7 macrophages stimulated with LPS *LRCH4* mRNA levels are downregulated (Fejes et al., 2018; Aloor et al., 2019). Microglia isolated from rat spinal cords after spinal cord injury (SCI) express lower levels of *LRCH1* mRNA and protein compared to microglia from sham-operated rats (Chen et al., 2020). In human primary macrophages, infection with *Staphylococcus aureus*, *Mycobacterium tuberculosis*, *Listeria monocytogenes* (*L. monocytogenes*), and enterohemorragic *E. coli* induces a downregulation of *LRCH4*, suggesting that LRCH proteins may function as critical effector molecules under inflammatory conditions, especially during host defense (Ng et al., 2011).

## Function of LRCH Proteins

### Cell Division

The first functional analyses of LRCH proteins focused on cell division and development in drosophila (Foussard et al., 2010). Here, dLRCH localizes at the cell cortex and cleavage furrow during mitosis of S2 cells and partly co-localizes with F-actin. Knockdown of dLRCH induces abnormal cortical protrusions and mis-positioning of the mitotic spindle but interferes only mildly with cell division. Similarly, knockdown of hLRCH3 in HeLa cells leads to cell division arrest and impairs chromosome segregation (Kittler et al., 2007). LRCH proteins seem to be regulated by phosphorylation as the phosphorylation patterns of hLRCH1, hLRCH3, and hLRCH4 in HeLa cells change between G_1_- and M-phase (Dephoure et al., 2008). In contrast, dLRCH-deficient flies develop normally indicating that cell division is not fatally affected *in vivo*. However, dLRCH knockout flies show a markedly impaired development when raised under low and high temperatures and a shortened life span, suggesting a role of dLRCH for fitness and aging (Foussard et al., 2010). A potential role of LRCH proteins during aging is further supported by transcriptome analyses of post-mortem brain and lymphocyte mRNA of humans, where *LRCH4* mRNA is increased with age (Hong et al., 2008). LRCH proteins are also linked to the regulation of cell proliferation on a molecular level (Gewurz et al., 2012). Here, LRCH3 plays a role in the activation of NFκB, a transcription factor critical for cell proliferation, survival and differentiation. Knockdown of LRCH3 increases IκBα levels and decreases NFκB activation in HEK293 cells when stimulated with tumor necrosis factor α (TNFα), interleukin 1β (IL-1β), or LMP1 (Gewurz et al., 2012).

### Immune Responses

A deeper understanding of LRCH protein function has been gained through research in leukocytes (Table 1). Here, LRCH proteins play essential roles in the innate as well as the adaptive immune system, regulating leukocyte migration and host defense. LRCH1 has been deciphered as a negative regulator of natural killer (NK) cell function (Dai et al., 2020). LRCH1-deficient NK92 cells show a higher cytotoxicity toward tumor cells, a higher secretion of interferon-γ, TNFα, IL-2 and higher granzyme B levels. Activation of NK cells and cytokine production is mediated by surface receptor NKp30 and activation of Src family kinases (SFKs) Src and Lck. In LRCH1-deficient NK92 cells phosphorylation of these SFKs is increased at basal levels and further increases in presence of tumor cells compared to control cells. Furthermore, LRCH1 negatively regulates activation and function of rat microglia via a p38 mitogen-activated protein kinase (MAPK) and ERK1/2-dependent pathway (Chen et al., 2020). Upon priming with LPS and ATP, LRCH1-knockdown microglia release higher levels of TNFα, IL-6 and IL-1β and develop into a pro-inflammtory phenotype. In rats injected with LRCH1-knockdown microglia, SCI results in higher neuronal loss and locomotor function impairment compared to SCI rats that have been injected with LRCH1-expressing microglia. In CD8^+^ T cells, LRCH1 binds linker for activation of T cells (LAT) thereby inhibiting recruitment of growth factor receptor-bound protein 2 to LAT and promotes LAT endocytosis and degradation, resulting in the termination of T cell receptor (TCR) signaling (Liu et al., 2020). Hence, LRCH1-deficient mice show improved clearance of infection with influenza and *L. monocytogenes* compared to WT mice and LRCH1-deficient cytotoxic T cells clear B16-MO5 tumor cells more efficiently than WT cells. A more direct regulation of the actin cytoskeleton by LRCH proteins is reported in CD4^+^ T cells (Xu et al., 2017; Wang et al., 2020). Here, LRCH1 has been identified as an inhibitory binding partner of DOCK8. Activation of DOCK8 leads to dissociation of the LRCH1-DOCK8 complex and activation of cell division control protein 42 homolog (Cdc42) by DOCK8 (Xu et al., 2017). Overexpression of LRCH1 or its DOCK8-binding domain (LRR_1__–__9_) reduces infiltration of the central nervous system by LRCH1-overexpressing CD4^+^ T cells in a model of experimental autoimmune disease (EAE). Accordingly, LRCH1-deficient CD4^+^ T cells present a more severe EAE phenotype due to increased Cdc42 activity and CD4^+^ T cell migration. Similarly, migration of CD4^+^ T cells from ulcerative colitis patients is increased compared to CD4^+^ T cells from healthy individuals (Wang et al., 2020). Here, CD4^+^ T cells mediate the pathogenesis of ulcerative colitis and massively infiltrate into the colonic mucosa, potentially by a mechanism negatively regulated by LRCH1 as migration of CD4^+^ T cells from ulcerative colitis patients is reduced by LRCH1 overexpression. These data indicate a regulation of the actin cytoskeleton by LRCH1-DOCK8 complex-dependent signaling. This is further supported by proteomic studies that have detected interactions between LRCH1/2/3/4 and DOCK6/7/8 (Couzens et al., 2013; Huttlin et al., 2017; O’Loughlin et al., 2018). Here, LRCH proteins interact with each other, with DOCK proteins and DOCK proteins with each other. Detailed analyses reveal that LRCH3 binds to DOCK7 via its LRR domain, to myosin-6 via a region between the LRR and CH domain and to actin-stabilizing septin proteins via the CH domain, inducing septin delocalization from actin filaments (O’Loughlin et al., 2018).

**TABLE 1:** Murine, rat, and human LRCH genes and proteins with a role in leukocyte function.

Species	Gene	Protein	Cell type	Function	References
Murine	*Lrch1*	LRCH1	T8.1 cells, primary CD4^+^ T cells	Negative regulation of Cdc42 via DOCK8 binding.	Xu et al., 2017
			Primary CD8^+^ T cells	Negative regulation of TCR signaling via LAT binding.	Liu et al., 2020
	*Lrch4*	LRCH4	RAW 264.7 cells	LPS binding and delivery to TLR4 in lipid rafts.	Aloor et al., 2019
Rat	*Lrch1*	LRCH1	Primary microglia	Negative regulation of p38 MAPK and Erk1/2 activation.	Chen et al., 2020
Human	*LRCH1*	LRCH1	Primary CD4^+^ T cells	Negative regulation of T cell migration via PKCα.	Wang et al., 2020
	*LRCH2*	LRCH2	NK92 cells	Negative regulation of SFK Src and Lck activity.	Dai et al., 2020
	*LRCH3*	LRCH3	Primary monocyte-derived DCs	Unknown. *LRCH3* downregulation upon LPS stimulation.	Ceppi et al., 2009
			Primary T cells	Negative regulation of proliferation, migration and interferon-γ production.	Liu et al., 2020
	*LRCH4*	LRCH4	Neutrophil-like HL-60 cells	Coro1A-interacting protein.	Pick et al., 2017
			Primary macrophages	Unknown. *LRCH4* downregulation upon bacterial infection.	Ng et al., 2011
			Primary monocyte-derived DCs	Unknown. *LRCH4* downregulation upon LPS stimulation.	Ceppi et al., 2009

In a sole study, Aloor et al. (2019) suggest LRCH4 to act mainly through its longest isoform harboring the TM domain. Here, LRCH4 is required for the capture of LPS at the plasma membrane and the transport into lipid rafts where LPS binds to TLR4 clusters (Aloor et al., 2019). Hence, LRCH4-deficient RAW 264.7 macrophages show reduced TNFα and granulocyte colony-stimulating factor levels upon LPS stimulation. Furthermore, stimulation of other TLRs, such as TLR7 and TLR9 also results in attenuated TNFα induction, indicating a broad spanning role for LRCH4 in TLR signaling. Moreover, LRCH4 has been identified as interacting partner of Coro1A, an actin-binding protein functionally linking β_2_ integrins to the actin cytoskeleton and therefore critical for β_2_ integrin activation controlling neutrophil adhesion, migration and phagocytosis (Yan et al., 2007; Pick et al., 2017). In summary, a large body of evidence suggests that LRCH proteins act as signaling or scaffold proteins regulating actin cytoskeleton dynamics, at least partially via an LRCH-DOCK axis.

## Discussion

To date, the precise function of LRCH proteins is still incompletely understood. Functional analyses have been almost absent until studies within the last decade uncovered a functional impact of LRCH proteins mainly in leukocytes. Nonetheless, several key questions have yet to be answered to improve our understanding of LRCH protein function in leukocytes. (1) Are LRCH proteins able to bind actin via the CH domain? LRCH proteins contain a single type 3 CH domain. There is an ongoing debate whether or not type 3 CH domains are able to bind actin as suggested for IQGAP1 (Mateer et al., 2004). As type 1 and type 2 CH domains act in tandem it is suggested for type 3 CH domain-containing proteins that they may form dimers, which has been reported in LRCH proteins recently, and thereby gain the ability to bind F-actin (Gimona and Mital, 1998; Pålsson-McDermott and O’Neill, 2007; Ng et al., 2011; Liu et al., 2020). However, a large body of evidence suggests that LRCH proteins regulate the cytoskeleton via interactions with cytoskeletal regulatory proteins, such as DOCK or septin proteins without direct binding to F-actin (Xu et al., 2017; O’Loughlin et al., 2018). (2) Is dimerization necessary for correct LRCH protein function or do LRCH proteins have diverse functions depending on their multimerization state? (3) Moreover, the function of TM domain-containing LRCH4 has been described in macrophages, nonetheless the question remains whether LRCH proteins that contain a TM domain mainly function at the plasma or also at inner membranes. (4) How do isoforms with and without TM domains differ in function? (5) It is also unclear how the four different LRCH proteins differ or overlap in their function. The sequence similarity within LRR and CH domains suggests that they have overlapping functions as reported for LRCH3 that can compensate for the loss of LRCH1 (Liu et al., 2020). They may also bear divergent functions due to their modest sequence similarity outside these domains. Hence, there is a need for studies comparing the effect of single and multiple knockouts to clarify this issue. (6) Currently, there is a lack of information on some leukocyte subsets, such as neutrophils and B cells. What is the function of LRCH proteins in these cell types? In T cells, LRCH1 negatively regulates TCR signaling which is similar to β_2_ integrins and B cell receptors, indicating a potential role of LRCH proteins in neutrophils and B cells (Mocsai et al., 2006). In conclusion, the current data on LRCH proteins indicate their importance for cell signaling and homeostasis, especially in leukocytes, and reveal that their dysregulation or absence results in inflammation and disease.

## Author Contributions

TR, AB, KP, BW, and DM-B wrote the manuscript. TR and DM-B prepared the table and figure. All authors contributed to the article and approved the submitted version.

## Conflict of Interest

The authors declare that the research was conducted in the absence of any commercial or financial relationships that could be construed as a potential conflict of interest.

## Footnotes

1 http://phobius.sbc.su.se
